# *Lactobacillus plantarum* Restores Intestinal Permeability Disrupted by *Salmonella* Infection in Newly-hatched Chicks

**DOI:** 10.1038/s41598-018-20752-z

**Published:** 2018-02-02

**Authors:** Lihong Wang, Long Li, Yan Lv, Qiaoling Chen, Junchang Feng, Xin Zhao

**Affiliations:** 1 College of Animal Science and Technology, Northwest A&F University, Yangling, China; 2 Department of Animal Engineering, YangLing Vocational & Technical College, Yangling, China; 3 Department of Animal Science, McGill University, Montreal, QC Canada

## Abstract

*Salmonella* infections in newly hatched chicks result in enteric and systemic diseases with a high mortality. Probiotics can improve the health of a host. The purpose of the present study was to investigate the effect of *Lactobacillus plantarum* LTC-113 on the gut permeability in the presence or absence of *Salmonella* (*Salmonella* Typhimurium) infection. Newly hatched chicks were randomly allocated to 4 treatments (i) NC (negative control); (ii) LAC (the *L. plantarum* LTC-113-treated group); (iii) SAL (the *Salmonella*-infected group), and (iv) LAC + SAL (the *L. plantarum* LTC-113-treated and *Salmonella*-infected group). Compared with the NC group, the intestinal permeability and claudin-2 (CLDN-2) were significantly increased, while mRNA levels of zonula occludens-1 (ZO-1) and claudin-5 (CLDN-5) were significantly decreased in the SAL group. However, these changes were eliminated in the LAC + SAL group. Additionally, numbers of *Salmonella* in liver, spleen and ceca were significantly reduced in the LAC + SAL group compared with the SAL group. Moreover, *L. plantarum* LTC-113 prevented the increase of inflammatory meditators myeloperoxidase (MPO), LITAF, IL-1β, IL-6 and inflammation scores induced by *Salmonella*. These findings indicate that *L. plantarum* LTC-113 can protect hosts from *Salmonella* induced intestinal barrier disruption by regulating expression of tight junction genes and inflammatory meditators and decreasing *Salmonella* colonization.

## Introduction

The genus *Salmonella* is one of the most common gastrointestinal pathogens in chickens. To date, more than 2000 serovars of *Salmonella* have been identified. Chickens can be colonized by numerous serovars of *Salmonella*. Among them, *S*. Pullorum and *S*. Gallinarum are specific for chickens. On the other hand, other serovars, such as *S*. Typhimurium, *S*. Enteritidis and *S*. Heidelberg, have a broad range of hosts and are most commonly associated with human infections^1^. While *S*. Typhimurium is not the most prevalent in poultry clinical cases, it is certainly one of the most important isolates. For example, Lamas *et al*.^2^ investigated the prevalence of *Salmonella* isolated from broiler houses between 2011 and 2015 and reported that S. Typhimurium was the most prevalent *Salmonella* among sixteen different serotypes found.

*Salmonella* cause over 90 million cases of Salmonellosis worldwide every year; about 85% of which are caused by *Salmonella* contaminated food^3^. In the United States, the total annual cost of foodborne salmonellosis was about $3.6 billion in 2013^4^. Poultry are considered one of the most important *Salmonella* reservoirs. Exposure to contaminated poultry-derived foods, mainly eggs and egg products, but also chicken meat, is one of the main reasons for Salmonellosis in humans. Therefore, preventing the *Salmonella* infection in poultry may be an effective measure to decrease salmonellosis in humans.

*Salmonella* infections occur via oral transmission where this pathogen must overcome several intestine barriers in the host to finally interact with the intestinal epithelium and thereby penetrate into deeper tissues. The intestine barriers include the luminal microbiota, a mucus layer, epithelial integrity and immune responses^5^. Oral infection of newly hatched chicks with *S*. Typhimurium was capable of inducing the expression of chemokines IL-8 and K60 along with the proinflammatory cytokine IL-1β in intestinal tissues and in the liver, indicative of an early inflammatory response^6^. However, such responses were not observed in 1 week old birds^7^. The different responses between newly hatched chicks and older birds could be mainly due to the following two reasons. Firstly, the gut microbiota in newly hatched chicks is going through a transition from a transient community to one of increasing complexity as the birds age. *Proteobacteria* are the predominant phylum in one-day old chicks, whereas *Firmicutes* are the predominant one in older birds (14–28 days of age). The latter also has a significantly more diverse microbial community structure^8^. Secondly, the gut-associated lymphoid tissue contains functionally immature T and B lymphocytes at the time of hatching, indicative of immature immunity of the newly hatched chicks^9^. Different cellular junctions, including tight junctions (TJ), adherence junctions (AJ), desmosomes and gap junctions, work together to maintain the integrity of the epithelial barrier^10^. Apical junctional complexes (AJC), consisting of TJ and AJ, are the most important in maintaining permeability of the epithelium by selectively restricting paracellular diffusion^11^. Previous studies have shown that broilers challenged with *S*. Typhimurium at 7 days of age exhibited decreased expression of CLDN-1, occludin, and mucin-2 transcripts at day21^12^. The *in vivo* results supported previous *in vitro* findings that *S*. Typhimurium decreased expression of CLDN-1, ZO-1, and E-cadherin and influenced the distribution of these proteins in Caco-2 cells^13–15^.

One important beneficial effect of probiotics is to enhance epithelial barrier functions^16^, through regulation of cellular junctions. Hummel *et al*.^11^ found that four *Lactobacilli*, including *L. acidophilus, L. fermentum*, *L. gasseri*, and *L. rhamnosus*, strongly increased the transepithelial resistance and modulated the gene expression of adherence junction proteins such as E-cadherin and β-catenin in T84 cells. Mennigen *et al*.^17^ found that the probiotic mixture VSL#3 ameliorated the leakiness of the colonic epithelium to Evans blue and prevented the decrease in expression and redistribution of the tight junction proteins occludin, ZO-1, and CLDN-1, -3, -4, and -5 in dextran sodium sulfate (DSS)-induced colitis mice. Another way by which probiotics enhance epithelial barrier functions is to inhibit the cell apoptosis. The probiotics mixture VSL#3 decreased apoptosis in a murine model of colitis^17^. Nevertheless, the effects of probiotics on the intestinal barrier function of newly hatched chickens challenged with *Salmonella* have not been reported. Therefore, we hypothesized that *L. plantarum* LTC-113 could restore the intestinal barrier disrupted by *Salmonella* infection in newly-hatched chicks. The purpose of the present study was to investigate the effect of *L. plantarum* LTC-113 on the gut permeability in the presence or absence of *Salmonella* infection.

## Results

### Colonization of ceca, spleen and liver by *S*. Typhimurium

**Figure 1:**
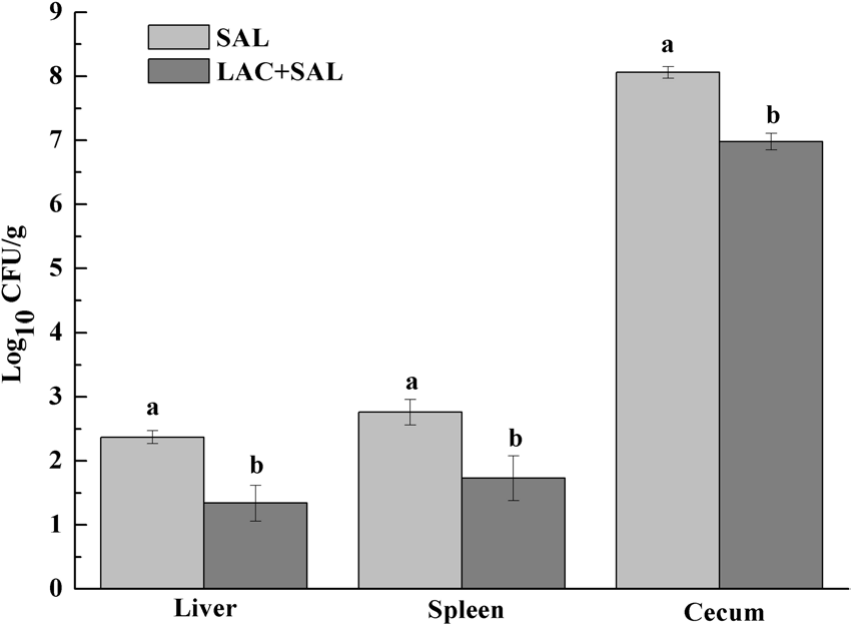
Numbers of *Salmonella* in liver, spleen and ceca of chicks in the SAL and LAC + SAL groups. SAL: the *Salmonella*-infected group; LAC + SAL: the *L. plantarum* LTC-113-treated and *Salmonella*-infected group. ^ab^Different letters mean significant differences between groups (*P* < 0.05, n = 6).

In order to determine the effect of the oral *Salmonella* challenge, the amounts of *S*. Typhimurium in ceca, spleen and liver were determined. *S*. Typhimurium was not detectable in the tissue samples from the NC and LAC groups. In comparison with the SAL group, the number of *S*. Typhimurium colonized in the liver, spleen and ceca were significantly lower in the LAC + SAL group (*P* < 0.05) (Fig. 1).

### Intestinal permeability

**Figure 2:**
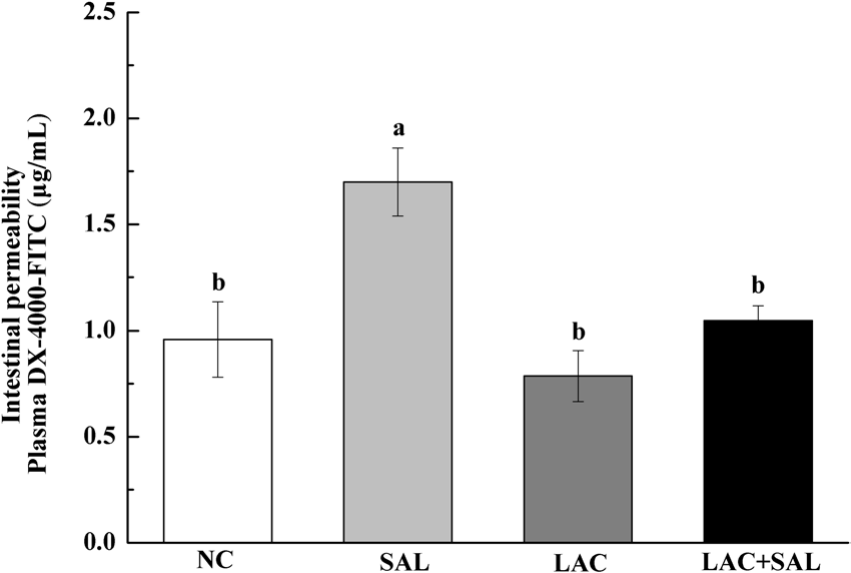
Effects of different treatments on the intestinal permeability in newly hatched chicks. Intestinal permeability was determined by measuring the DX-4000-FITC level in plasma. NC, the negative control, no *L. plantarum* LTC-113 treatment, no *Salmonella* infection; SAL, the *Salmonella*-infected group; LAC, the *L. plantarum* LTC-113-treated group; LAC + SAL, the *L. plantarum* LTC-113-treated and *Salmonella*-infected group. ^ab^Different letters mean significant differences (*P* < 0.05, n = 5).

To determine whether translocation of *S*. Typhimurium into spleen and liver was associated with altered intestinal permeability, intestinal paracellular permeability was determined by measuring DX-4000-FITC in plasma. As showed in Fig. 2, *S*. Typhimurium infection significantly increased the intestinal permeability as compared with the NC group (*P* < 0.05). However, the intestinal permeability of chicks in the LAC + SAL group was significantly lower than that in the SAL group (*P* < 0.05). There was no significant difference between the LAC group and the LAC + SAL group (*P* > 0.05).

### Expression of AJ and TJ genes

**Figure 3:**
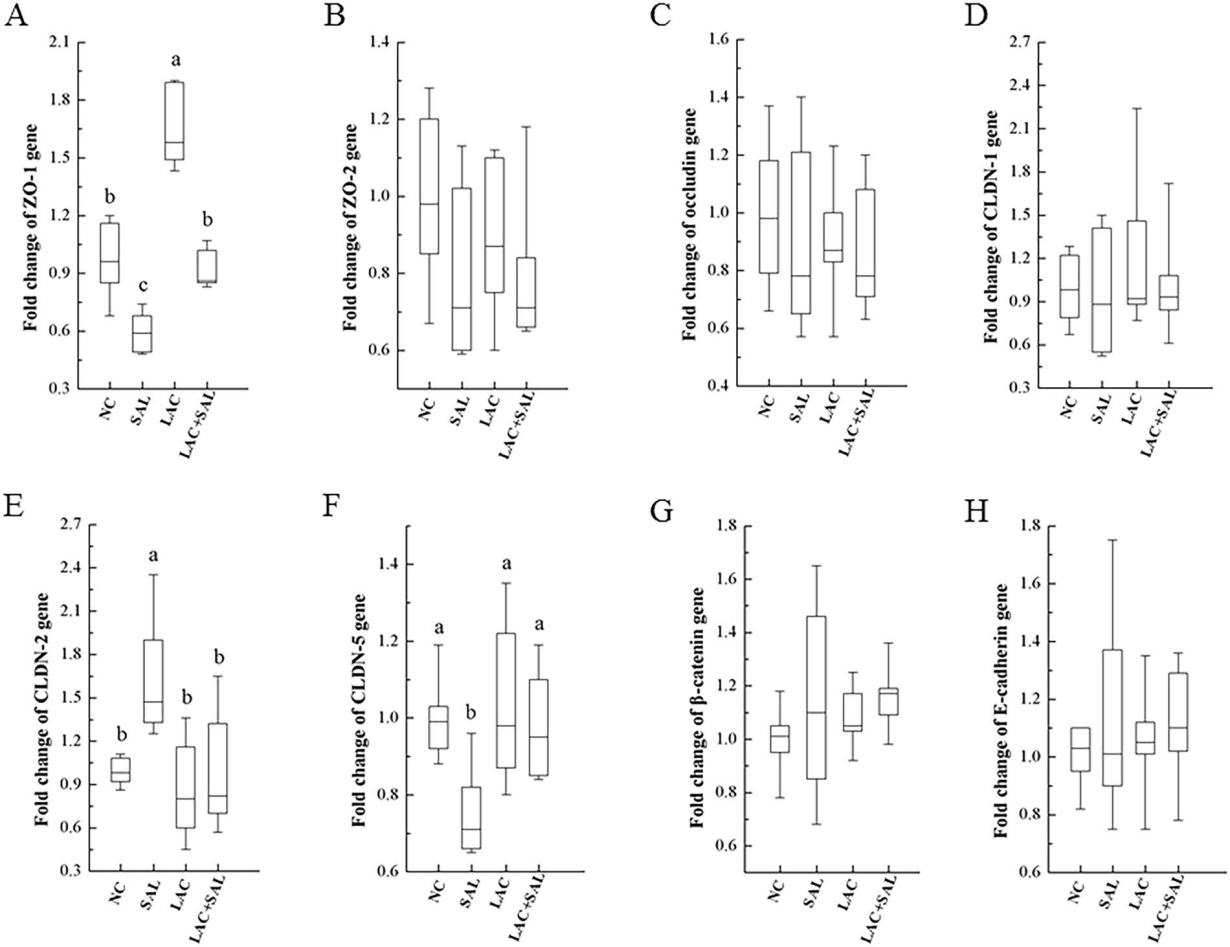
Relative expression levels of TJ and AJ associated genes in the ceca of newly hatched chicks. NC, the negative control, no *L. plantarum* LTC-113 treatment, no *Salmonella* infection; SAL, the *Salmonella*-infected group; LAC, the *L. plantarum* LTC-113-treated group; LAC + SAL, the *L. plantarum* LTC-113-treated and *Salmonella*-infected group. ^abc^Different letters mean significant differences among groups (*P* < 0.05, n = 6). Box plots show the fold change of genes. Boxes extend from the third quartile (Q3) to first quartile (Q1), with the line at the median.

In order to investigate why the intestinal permeability was changed by *S*. Typhimurium and *L. plantarum* LTC-113, expression of selected AJ and TJ genes was measured by RT-PCR. As showed in Fig. 3, there was no significant difference in occludin, β-catenin, E-cadherin, CLDN-1 and ZO-2 at the mRNA level among all four groups (*P* > 0.05). In comparison with the NC group, *Salmonella* infection (the SAL group) significantly decreased mRNA levels of ZO-1 and CLDN-5 and increased the mRNA level of CLDN-2 (*P* < 0.05). The down-regulation of ZO-1 and CLDN-5 and the up-regulation of CLDN-2 due to *Salmonella* infection were eliminated in the LAC + SAL group. Moreover, the mRNA expression level of ZO-1 in the LAC group was significantly higher than that in the NC group (*P* < 0.05) (Fig. 3).

### Intestinal histopathology and cell apoptosis analysis

**Figure 4:**
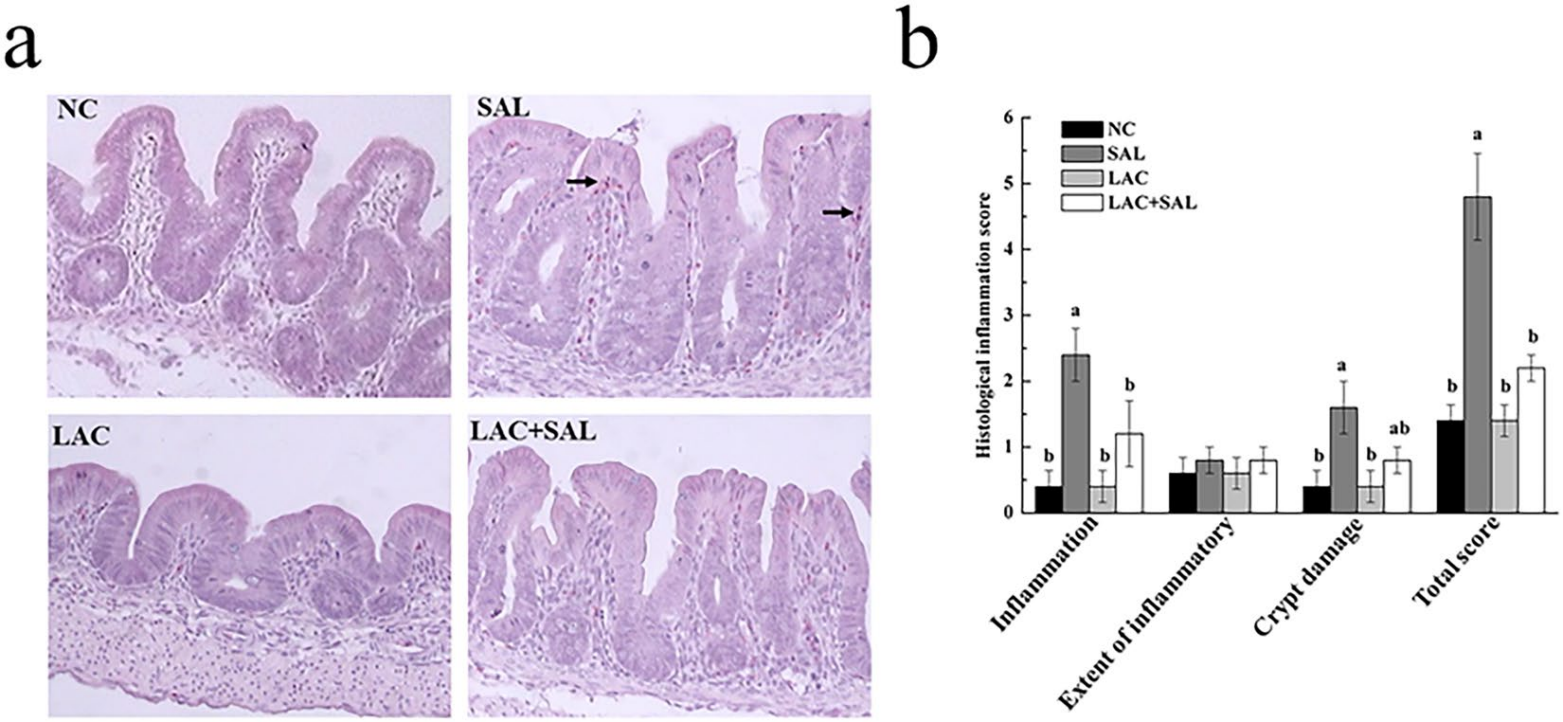
Effects of different treatments on histopathology (**a**) and histological inflammation scores (**b**) of ceca in newly hatched chicks. NC, the negative control, no *L. plantarum* LTC-113 treatment, no *Salmonella* infection; SAL, the *Salmonella*-infected group; LAC, the *L. plantarum* LTC-113-treated group; LAC + SAL, the *L. plantarum* LTC-113-treated and *Salmonella*-infected group. Arrows indicate heterophils. In the SAL group, a few heterophils were found and intestinal villus were swelled and arranged irregularly. In the LAC + SAL group, inflammation was less severe, and heterophils were not founded. ^ab^Different letters mean significant differences among groups (*P* < 0.05, n = 6).

**Figure 5:**
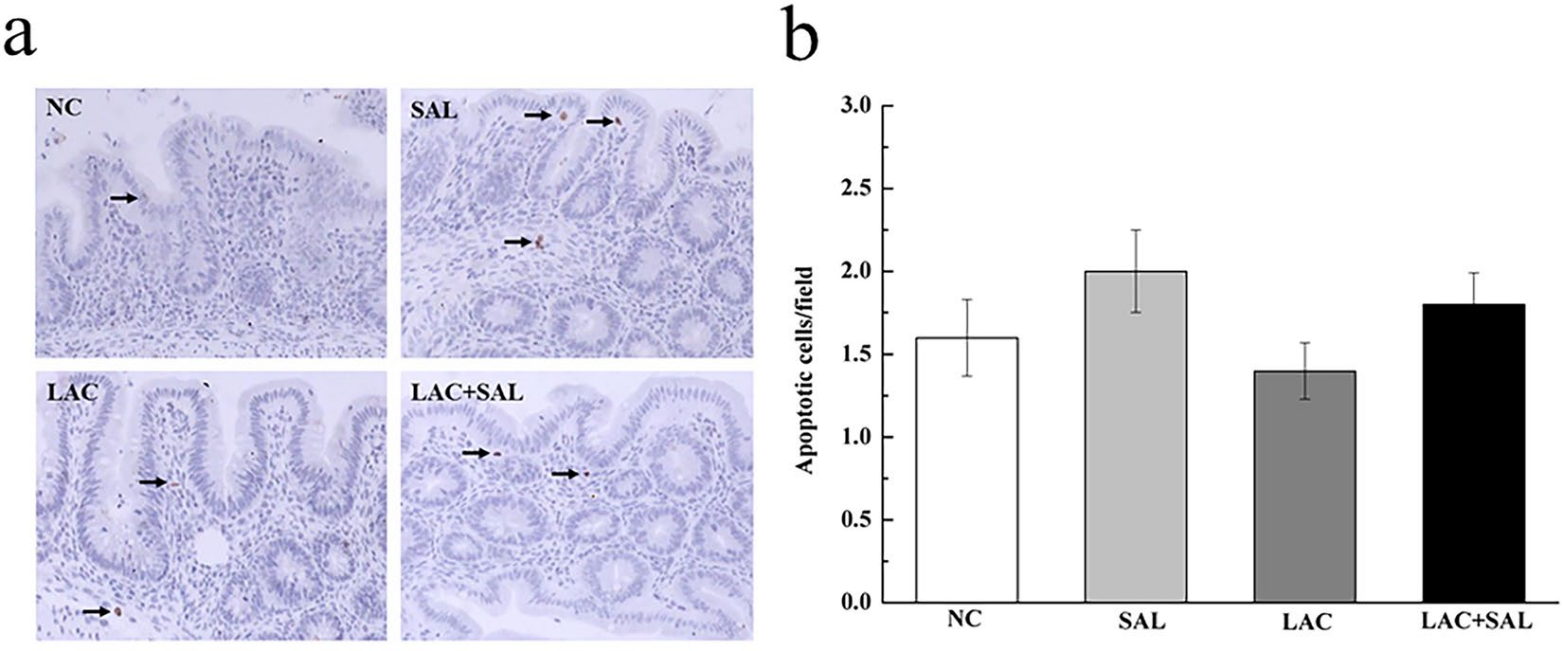
Effects of different treatments on intestinal cell apoptosis of ceca in newly hatched chicks. (**a**) TUNEL assay was used for *in situ* detection of cell apoptosis; (**b**) the average number of apoptotic cells was counted in each field. NC, the negative control, no *L. plantarum* LTC-113 treatment, no *Salmonella* infection; SAL, the *Salmonella*-infected group; LAC, the *L. plantarum* LTC-113-treated group; LAC + SAL, the *L. plantarum* LTC-113-treated and *Salmonella*-infected group. Arrows indicate TUNEL-positive cells. There were a few TUNEL-positive cells in every group.

The morphology of cecal samples was monitored to investigate whether *S*. Typhimurium infection affected the intestinal integrity. A small number of heterophils were found only in the SAL group, indicating a mild inflammatory reaction at 8 hpi (Fig. 4a). Moreover, SAL strongly increased the inflammation score, crypt damage score and total score (*P* < 0.05). *L. plantarum* LTC-113 pretreatment (LAC + SAL) significantly decreased the inflammation score and total score compared with the SAL group (*P* < 0.05), but the crypt damage score was not affected by *L. plantarum* LTC-113 (*P* > 0.05) (Fig. 4b). Apoptotic cells were found in all treatment groups at 8 hpi (Fig. 5a). However, there were no significant differences in apoptosis rates at 8 hpi among the four treatment groups (*P* > 0.05) (Fig. 5b).

### The activity of myeloperoxidase (MPO) in serum

**Figure 6:**
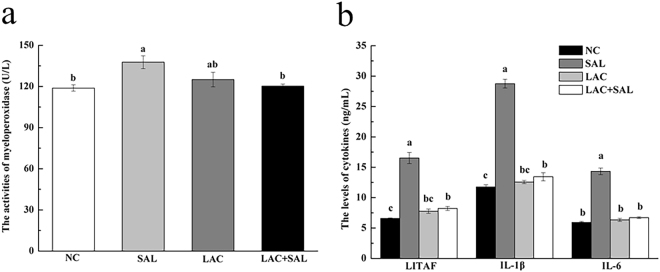
Effects of different treatments on activities of MPO (**a**) and levels of cytokines (**b**) in serum. NC, the negative control, no *L. plantarum* LTC-113 treatment, no *Salmonella* infection; SAL, the *Salmonella*-infected group; LAC, the *L. plantarum* LTC-113-treated group; LAC + SAL, the *L. plantarum* LTC-113-treated and *Salmonella*-infected group. ^abc^Different letters mean significant differences among groups (*P* < 0.05, n = 6).

To evaluate the intestinal inflammation, levels of an inflammatory mediator MPO in serum samples were determined. The MPO activities of chickens in the SAL group were significantly higher than in the NC group (*P* < 0.05). In comparison with the SAL group, *L. plantarum* LTC-113 pretreatment (LAC + SAL) significantly decreased the activities of MPO (*P* < 0.05) (Fig. 6a).

### Assay of the cytokines LITAF, IL-1β, and IL-6 in serum

To further study inflammatory responses induced by *Salmonella* infection, levels of cytokines LITAF, IL-1β, and IL-6 in serum samples were determined by ELISA (Fig. 6b). *Salmonella* infection (SAL group) significantly increased the levels of LITAF, IL-1β, and IL-6 in serum compared with the NC group (*P* < 0.05). However, the levels of LITAF, IL-1β, and IL-6 in the LAC + SAL group were significantly lower than in the SAL group (*P* < 0.05).

## Discussion

Chicks at one day old were used in this study, since they have a low diversity and density of gut microbiota and an immature immunity. *Salmonella* easily colonize and act on the intestinal epithelium of newly hatch chicks. The gut of a chick during the first 3 days is only protected by high expression of β-defensins^18^. An activation and normalization of the innate immune system, characterized by high expression of cytokines IL-8 and IL-17, starts on day 4 post-hatch^18^.

To the best of our knowledge, this study is the first to evaluate the effect of *S*. Typhimurium infection on the epithelial integrity of newly hatched chicks by measuring permeability of DX-4000-FITC. In this study, *Salmonella* infection was accompanied by a loss of epithelial integrity, as indicated by increased permeability of DX-4000-FITC. Zhang *et al*.^12^ reported that *S*. Typhimurium infection caused intestinal injury in broiler chickens, as determined by an increase in plasma endotoxin levels. Moreover, disruption of the intestinal epithelial barrier by S. Typhimurium was also reflected by translocation of *S*. Typhimurium into spleen and liver in our current study. Our result was in agreement with the studies of Zhang *et al*.^19^ and Feng *et al*.^20^ in chicks. In addition, Köhler *et al*.^13^ found that infection of human intestinal T84 cells by *S*. Typhimurium over 2 h resulted in an 80% loss of transepithelial electrical resistance, increased bacterial translocation and initiation of polymorphonuclear leukocyte migration across the intestinal barrier and increased the paracellular influx of inulin. Similarly, Yu *et al*.^15^ found that *S*. Typhimurium SL1344 increased the dextran permeability and decreased the transepithelial electrical resistance in Caco-2 cells. Streptomycin-pretreated mice challenged with *Salmonella* resulted in translocation of *Salmonella* into the liver^21^. Li *et al*.^22^ also found that *Salmonella* damaged intestinal structure severely of mice and resulted in the increase of bacteria translocation into the mesenteric lymph node. These results demonstrate that *Salmonella* infections can disrupt the intestinal permeability of hosts.

The paracellular pathway of intestinal cell is mainly modulated by cell junction complexes TJ and AJ^11^. Both junction complexes are formed by various transmembrane proteins. The core of TJ complexes is composed of occludin, ZO and CLDN proteins, while the core of AJ complexes is composed of β-catenin and E-cadherin. Therefore, to better clarify the molecular mechanism of *S*. Typhimurium induced loss of intestinal epithelial barrier functions, changes in the gene expression of 5 transmembrane proteins (occludin, CLDN-1, CLDN-2, CLDN-5 and E-cadherin) and 3 intracellular proteins (ZO-1, ZO-2, β-catenin) of AJ and TJ at the mRNA level were determined.

Our results showed that *S*. Typhimurium infection up-regulated the gene expression of CLDN-2 and down-regulated the gene expression of ZO-1 and CLDN-5. Claudins are mainly responsible for linking adjacent enterocytes through interactions of their extracellular loops in TJ complexes. CLDN-5 is known to form tight junctions and decrease permeability, whereas CLDN-2 stimulates expression of cation-selective pores and increases the permeability^23^. The ZO-1 scaffolding proteins play an important role in linking transmembrane junctional proteins to the actomyosin cytoskeleton and several cytoplasmic regulatory proteins^24^. In this study, an increase in CLDN-2 and reductions in the expression of ZO-1 and CLDN-5 induced by *S*. Typhimurium disrupted the intestinal barrier, consequently enabling *S*. Typhimurium translocation into the spleen and liver. Köhler *et al*.^13^ reported that T84 monolayers infection with *S*. Typhimurium dramatically decreased expression of ZO-1 and led to loss of the barrier function. Yu *et al*.^15^ and Shao *et al*.^25^ also reported that *Salmonella* infection decreased expression of ZO-1 protein and caused a large increase in permeability in Caco-2 cells. Zhang *et al*.^19^ found that *Salmonella* infection increased the leaky protein CLDN-2, permeability and bacterial translocation in the human intestinal epithelial cell lines SKCO15 and HT29C19A as well as in streptomycin-pretreated C57BL6 mice. Moreover, studies have shown that TNF-α, and ILs also could regulate the intestinal barrier^26^. Cui *et al*.^27^ found that TNF-α could decrease the level of phosphorylation of CLDN-1, and dissociate CLDN-1 from TJs. TNF-α has not been found nor described in the broiler genome. However, LITAF is responsible for the expression of TNF-α in mammals^28^ and has been reported to play an important role in intestinal inflammation of broilers^29^. Suzuki *et al*.^30^ reported that IL-6 could cause the upregulation of CLDN-2, thus increasing mucosal permeability. In this study, *Salmonella* elevated the levels of LITAF and IL-6, this may be associated with the increase of permeability induced by *Salmonella*. Taken together, these results indicated that *S*. Typhimurium induced the increase of permeability by disrupting the structure of TJ and AJ complexes.

Besides changes in tight junction complexes, enhanced cell apoptosis is also associated with disruption of epithelial integrity^31^. In our study *Salmonella* infection did not cause an increase in intestinal cell apoptosis. This result disagrees with previous reports, possibly due to the fact that only the first 8 hours of *Salmonella* infection was studied. Santos *et al*.^32^ found that healthy male rhesus macaques challenged with *Salmonella* resulted in an increase in intestinal mucosa cell death. In an *in vitro* study, Li *et al*.^33^ reported that apoptosis was induced by *Salmonella* 12 hours after infection in Caco-2 cells. Paesold *et al*.^34^ found that apoptosis of epithelial cells infected with *Salmonella* was delayed for approximately 28 h after bacterial entry. Taken together, all the results suggest that *Salmonella* infection disrupts epithelial integrity mainly by interfering with the TJ and AJ complexes at the early stage and by apoptosis at the later stage.

Probiotics have been defined as living bacteria that, when ingested in sufficient quantity, improve the health of the host. Studies *in vitro* on epithelial monolayers and *in vivo* rat models have demonstrated that probiotics could improve barrier function^11,17^. In this study, administration of *L. plantarum* LTC-113 abolished the change of ZO-1, CLDN-5, and CLDN-2 induced by *Salmonella*. Meanwhile, *L. plantarum* LTC-113 eliminated the increased permeability and bacterial translocation induced by *Salmonella*. Qin *et al*.^35^ found that *Lactobacillus plantarum* could alleviate *Salmonella*-induced increase of dextran permeability and decrease of ZO-1 proteins in Caco-2 cells. Yu *et al*.^15^ found that *Lactobacillus. amylophilus* D14 protected Caco-2 cells from *S*. Typhimurium SL1344 induced increase of dextran permeability and decrease of ZO-1 proteins in Caco-2 cells. All above results indicate that *L. plantarum* LTC-113 protected the intestinal epithelial barrier from *Salmonella* infection through regulating expression of TJ and AJ proteins. In addition, the number of *Salmonella* in the intestinal was reduced by administration of *L. plantarum* LTC-113 in *Salmonella* infection chicks, which could be another mechanism by which *L. plantarum* LTC-113 protected the intestinal barrier integrity.

The *Salmonella* infection induced the damage of intestinal morphology in this study. The inflammation scores were elevated by *Salmonella* infection and *L. plantarum* LTC-113 could alleviate the damage of intestinal morphology and decrease inflammation scores. This result was accompanied by the change of myeloperoxidase (MPO) and proinflammatory cytokines LITAF, IL-1β, and IL-6. MPO is a leukocyte-derived enzyme and is an important indicator of inflammatory responses. In the present study, the increase in serum MPO activities of the SAL group suggested that *Salmonella* infection could activate monocytes and neutrophils in blood and promote intestinal inflammations. However, the decrease of MPO activities in the LAC + SAL group demonstrated that *L. plantarum* LTC-113 could depress *Salmonella* induced inflammatory responses. This was also supported by the results of cytokines LITAF, IL-1β, and IL-6. LITAF of chickens^29^. IL-1β is indicative of inflammation and is mainly secreted by monocytes, tissue macrophages and other cells^36^. IL-6 is associated with secretion of acute phase proteins that are involved in inflammatory responses^37^. In this study, *L. plantarum* LTC-113 prevented the increase of proinflammatory cytokines induced by *Salmonella* infection. Feng *et al*.^20^ have also demonstrated similar changes. Our results demonstrate that *Salmonella* infection could induce intestinal inflammation of newly hatched chicks by increasing the levels of inflammatory meditator myeloperoxidase and proinflammatory cytokines LITAF, IL-1β, and IL-6, and *L. plantarum* LTC-113 could alleviate intestinal inflammation induced by *Salmonella* in newly hatched chicks.

Resistance to colonization of *Salmonella* by the gut microbiota and the innate immune system are important for a host to prevent *Salmonella* from interacting with intestinal epithelium^38^. The gut microbial composition of young children continues to develop until 3 years of age^39^. Moreover, infants are more susceptible to *Salmonella* infections, due to the immaturity of their immune systems. These two phenomena exist in other young animals, especially in newly hatched chickens. In commercial poultry production, hatching is carried out in a clean incubator. Newly hatched chicks, unlike other mammals, will not have an access to maternal antibodies and healthy microbiota via contact with adult chickens. Consequently, the gut microbiota in newly hatched chickens is under-developed as indicated by low diversities and densities^40–42^. Moreover, the immunity of the newly hatched chicken is immature^9^. In this study, we demonstrate that *S*. Typhimurium infections could disrupt intestinal permeability and induce cecal inflammation. Thus, newly hatched chicks may be used as another model for studying *Salmonella* infections in young children.

In summary, *Salmonella* disrupted the intestinal epithelial barrier in newly hatched chicks by increasing expression of CLDN-2 and decreasing expression of ZO-1 and CLDN-5, thereby allowing bacterial translocation. Moreover, *L. plantarum* LTC-113 could protect newly hatched chicks from *Salmonella* induced intestinal epithelial barrier disruption by stabilizing the expression of tight junction genes, regulating the levels of inflammatory meditators, and decreasing the *Salmonella* colonization.

## Materials and Methods

### Chicks and experimental design

Eighty 1-day-old healthy male Nick chicks were purchased from a local hatchery (DaCheng hatchery, Xianyang, Shaanxi, China). The chicks were randomly divided into four groups. The treatment groups were as follows: (i) the negative control (no *L. plantarum* LTC-113 treatment and no *Salmonella* infection, NC); (ii) the *L. plantarum* LTC-113-treated group (10^9^ CFU *L. plantarum* LTC-113, LAC); (iii) the *Salmonella*-infected group (10^9^ CFU *S*. Typhimurium CVCC542, SAL); and (iv) the *L. plantarum* LTC-113-treated and *Salmonella*-infected group (10^9^ CFU *L. plantarum* LTC-113 and 10^9^ CFU *S*. Typhimurium CVCC542, LAC + SAL). All chicks had free access to water and a starter feed without antibiotics during the experiment. All experimental protocols used in this experiment were in accordance with those approved by the Northwest Agriculture and Forestry (A&F) University Institutional Animal Care and Use Committee (protocol number NWAFAC1036) and the institutional safety procedures were followed.

### Bacterial isolates, culture media, and growth conditions

The potential probiotic strain used in this study was isolated by our lab. Briefly, lactic acid bacteria (LAB) were isolated from intestinal contents of Tibet chickens and confirmed by sequencing of 16 s rRNA and comparison with the GenBank database. Among 139 isolated lactic acid bacteria strains, LTC-113 strain (*L. plantarum*) was selected based on preliminary *in vitro* and *in vivo* results for this study. To prepare the LAB inoculum, *L. plantarum* strain LTC-113 was propagated twice in the DeMan, Rogosa, and Sharpe (MRS) broth at 37 °C without shaking. The number of colony-forming units (CFU) in culture was measured by plating on MRS plates after a series dilution.

A spontaneous novobiocin-resistant of *S*. Typhimurium CVCC542 was obtained from the China Veterinary Culture Collection Center (Beijing, China). *S*. Typhimurium CVCC542 was grown overnight in the Luria-Bertani (LB) broth at 37 °C in an orbital shaking incubator at 180 rpm/min, sub-cultured twice, and then the CFU was measured by plating on LB plates after a series dilution.

### *L. plantarum* LTC-113 treatment and *Salmonella* infection

On the first day of age, chicks in groups LAC, SAL and LAC + SAL received 0.2 ml phosphate-buffered saline (PBS) containing 1 × 10^9^ CFU of the *L. plantarum* LTC-113, 1 × 10^9^ CFU of the *S*. Typhimurium CVCC542, 1 × 10^9^ CFU of the *L. plantarum* LTC-113 and 1 × 10^9^ CFU of the *S*. Typhimurium CVCC542 via oral gavage, respectively. The chicks in the NC group received 0.2 ml of sterile PBS.

### Tissue collection and storage

Eight hours after the *Salmonella* infection, 6 chicks from each group were randomly selected. Blood samples were collected from the carotid vein. For the separation of blood sera, blood samples were incubated for 1 h at room temperature followed by centrifuging at 2,000 g for 10 min. After blood collection, chickens were euthanized by cervical dislocation. One arm of cecum, liver and spleen were collected for bacterial enumeration. Meantime, a segment (2 cm) from one arm of the cecum (adjacent to cecal tonsils) was collected and separated into two parts. One part was fixed in 4% paraformaldehyde, while the other part was kept in liquid nitrogen for RNA extraction.

### Enumeration of *Salmonella*

The collected tissue samples (ceca, liver and spleen) were homogenized in sterile PBS and plated out onto the selective Brilliant Green agar containing 50 μg of novobiocin per ml after a series dilution. Plates were incubated at 37 °C for 24 h before enumeration of the colonies. All microbiological analyses were performed in duplicate and the average numbers of CFU were subjected to logarithmic transformation before statistical analysis. The results were calculated and expressed as log10 colony-forming units per gram tissue.

### Intestinal permeability

The intestinal permeability was measured by using 4000 Da fluorescent dextran–FITC (DX-4000–FITC) (FD4000, Sigma-Aldrich, Missouri, USA) as previously described^43^. Briefly, 5 chicks that had fasted for 4 h were randomly selected from each group and were given DX-4000–FITC (dissolved in sterile PBS, 125 mg/ml) by oral gavage (0.5 mg/g body weight) 8 hours post-infection (hpi). Our preliminary experiment indicated that *S*. Typhimurium infections induced the highest intestinal permeability of newly hatched chickens at 8 hpi. After 1 h, blood was collected from the carotid vein and was centrifuged at 4 °C, 2, 000 g for 10 min. Fluorescence intensity of 100 μL plasma was measured with a fluorescence spectrophotometer (Lambda25, Perkin Elmer, Wellesley, Massachusetts, USA) at an excitation wavelength of 485 nm and an emission wavelength of 535 nm. The concentrations of DX-4000-FITC per mL of sera were calculated from a standard curve with known FITC concentrations.

### Real-time qPCR

Total RNA from ceca was extracted by using the Takara Total RNA Kit I (Takara, Dalian, China) according to the manufacturer’s instructions and quantified by spectrophotometry (Nanodrop ND-1000, Thermo Scientific, Wilmington, USA). First-strand cDNA synthesis was performed using a reverse transcription kit (Takara, Dalian, China) and 1 μg of extracted total RNA according to the manufacturer’s instructions. The obtained cDNA was stored at −80 °C until use. Quantifications of target genes occludin, CLDN-1, CLDN-2, CLDN-5, ZO-1, ZO-2, E-cadherin, β-catenin and a housekeeping gene (glyceraldehyde-3-phosphate dehydrogenase (GAPDH)) in cDNA samples were carried out by fluorometric real-time PCR using a Bio-Rad CFX-96 instrument (California, USA) and real-time PCR kits (Takara, Dalian, China). Primers for specific genes were either described previously^44,45^ or newly designed with the Primer premier 5.0 software (Table 1). The reaction mixture for the qPCR contained 1 μL of the cDNA, 12.5 μL SYBR Premix Ex Taq (Takara, Dalian, China), 0.5 μL forward and reverse primers (final concentration of 0.4 μmol/mL for each primer) and 10.5 μL sterile water according to the manufacturer’s instructions. Each sample was analyzed in triplicate. Five housekeeping genes were tested after which the most stable housekeeping gene for cecal samples was selected, using the geNorm software (http://medgen.ugent.be/~jvdesomp/genorm/). GAPDH was finally chosen to normalize gene expression due to its high expression stability. The efficiency of all tested genes was between 90% and 110%. Target gene expression was normalized with GAPDH gene expression. The method of 2^−ΔΔCt^ was used to analyze the real-time PCR data^46^ and results were expressed as the fold change relative to the average value of the negative control group.

**Table 1:** Primers used to analyze gene expression by quantitative RT-PCR.

Gene	Primer sequence (5′–3′)	Size (bp)	Annealing temperature (°C)	Reference	GenBank No.
Occludin	Forward: ACCCCGAGTTGGATGAGT Reverse: CTTCCGAAAATCCCAATG	192	55	This study	NM_205128.1
CLDN-1	Forward: CTGATTGCTTCCAACCAG Reverse: CAGGTCAAACAGAGGTACAAG	140	59	ref.^44^	NM_001013611
CLDN-2	Forward: CCTCAGCCCTCCATCAAA Reverse: CTGCGTCTTCTCCTCTTACTGT	164	56	This study	NM_001277622.1
CLDN-5	Forward: CATCACTTCTCCTTCGTCAGC Reverse: GCACAAAGATCTCCCAGGTC	111	59	ref.^44^	NM_204201
ZO-1	Forward: CTTCAGGTGTTTCTCTTCCTCCTC Reverse: CTGTGGTTTCATGGCTGGATC	131	59	ref.^44^	XM_413773
ZO-2	Forward: CGGCAGCTATCAGACCACTC Reverse: CACAGACCAGCAAGCCTACAG	87	64	ref.^44^	NM_204918
E-cadherin	Forward: TCACGGGCAGATTTCTAT Reverse: CACGGAGTTCGGAGTTTA	133	57	ref.^45^	NM_001039258.1
β-catenin	Forward: CTGTTCAGAATGTCGGAGGA Reverse: CTGGGCACCAATGTCAAG	135	55	This study	NM_001277622.1
GAPDH	Forward: TGGAGAAACCAGCCAAGTAT Reverse: GCATCAAAGGTGGAGGAAT	145	55	This study	NM_204305.1

### Histopathology and TUNEL staining

Fragments from the cecum were fixed by immersion in the 4% buffered formalin for 24 hours, then rinsed with PBS and embedded in paraffin. The tissue was consecutively cut into 5 μm thickness sections and stained with hematoxylin and eosin for histopathological analyses (magnification × 40). Histological inflammation was graded by two blinded investigators using a score system introduced by Mennigen *et al*.^17^, including the degree of inflammation, the transmural vertical extent of inflammation and the crypt damage score, in relation to the percentage of involvement of mucosal surface in each slide (Table 2).

**Table 2:** Calculation of histological inflammation score.

Feature graded	Grade	Description
Inflammation	0	None
	1	Slight
	2	Moderate
	3	Severe
Extent	0	None
	1	Mucosa
	2	Mucosa and submucosa
	3	Transmural
Crypt damage	0	None
	1	Basal 1/3 damaged
	2	Basal 2/3 damaged
	3	Only surface epithelium intact
	4	Entire crypt and epithelium lost
Percent involvement	1	1–25%
	2	26–50%
	3	51–75%
	4	76–100%

A terminal transferase dUTP nick end labeling (TUNEL) assay was used for *in situ* detection of cell apoptosis. Five-micrometer sections of the cecum were de-paraffinized, hydrated, and treated with a solution containing proteinase K (20 mg/ml) and 0.5% Triton X-100 (Sigma, USA). *In situ* detection of cell apoptosis was performed using a commercial kit (DeadEnd™ Colorimetric TUNEL System, Promega, USA) according to the manufacturer’s instructions. Sample slides as positive controls were treated with RNase-free DNase I at room temperature for 10 min before incubation with the TUNEL reagent. Sample slides as negative controls were incubated with the TUNEL reagent in the absence of terminal deoxynucleotidyl transferase. At least 10 fields were selected randomly per animal and the number of apoptotic cells was counted in each field. The results were expressed as an average number of apoptotic cells in ten fields.

### Serum Parameters Measurement

The activity of myeloperoxidase (MPO) was determined according to the procedure of Wu *et al*.^47^ using a commercial kit (Jiancheng Bioengineering Institute, Nanjing, China). The activities of MPO in serum samples were expressed as U/L. The cytokines LITAF, IL-1β, and IL-6 in serum were measured by the double-antibody sandwich ELISA method with specific antibodies of LITAF, IL-1β, and IL-6 prepared by Feng *et al*.^20^ in our laboratory. Briefly, serum samples were added to each well and rabbit anti-chicken IL-6, LITAF, and IL-1β antibodies were applied as capturing antibodies. The chemical 3,3′,5,5′-Tetramethylbenzidine (TMB) was used as a chromogenic substrate and the color reaction was stopped by sulfuric acid solution. The absorbance values of well plates were read at 450 nm wavelength. The levels of cytokines in serum samples were determined by specific standard curves.

### Data Analysis

The data from the experiment were subjected to ANOVA after determination of variance homogeneity by using the SPSS 16.0 software. In this study, gene expression was subjected to the non-parametric Kruskal-Wallis analysis, followed by a Mann-Whitney test and the data are expressed as median and interquartiles. The tests for other parameters were performed using Duncan’s test for multiple comparisons. Differences are considered statistically significant at the level of *P* < 0.05 and data are presented as means ± SEM.
